# Experimental and Analytical Approaches for Improving the Resolution of Randomly Barcoded Transposon Insertion Sequencing (RB-TnSeq) Studies

**DOI:** 10.1021/acssynbio.2c00119

**Published:** 2022-06-03

**Authors:** Andrew J. Borchert, Alissa Bleem, Gregg T. Beckham

**Affiliations:** † Renewable Resources and Enabling Sciences Center, National Renewable Energy Laboratory, Golden, Colorado 80401, United States; ‡ Center for Bioenergy Innovation, Oak Ridge National Laboratory, Oak Ridge, Tennessee 37830, United States

**Keywords:** transposon insertion sequencing, baseline selection, *Pseudomonas putida*, gene function, data resolution

## Abstract

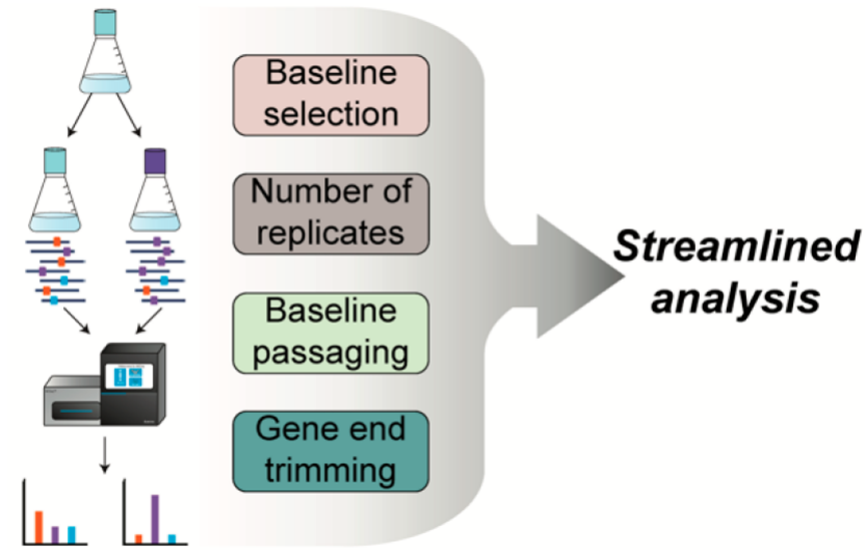

Randomly barcoded transposon insertion sequencing (RB-TnSeq) is an efficient, multiplexed method to determine microbial gene function during growth under a selection condition of interest. This technique applies to growth, tolerance, and persistence studies in a variety of hosts, but the wealth of data generated can complicate the identification of the most critical gene targets. Experimental and analytical methods for improving the resolution of RB-TnSeq are proposed, using *Pseudomonas putida* KT2440 as an example organism. Several key parameters, such as baseline media selection, substantially influence the determination of gene fitness. We also present options to increase statistical confidence in gene fitness, including increasing the number of biological replicates and passaging the baseline culture in parallel with selection conditions. These considerations provide practitioners with several options to identify genes of importance in TnSeq data sets, thereby streamlining metabolic characterization.

Transposon insertion sequencing (TnSeq) is a widely used systems biology tool that combines the broad genomic modification capability of transposons with the throughput power of next generation sequencing to characterize gene function,^1^ as employed for catabolism,^2^ virulence,^3,4^ stress resistance,^5,6^ and environmental adaptation.^7,8^ When the transposon insertion density sufficiently saturates the genome, genes contributing to function(s) of interest are identified by enumerating strains before and after a desired growth selection.^9^ The resulting “gene fitness”, roughly the log_2_ ratio of insertion mutant abundance after vs before selection, quantifies the relative importance of each gene, with greater fitness amplitude indicating greater importance for growth under a given selection. More recently, a strategy termed randomly barcoded transposon insertion sequencing (RB-TnSeq) emerged, which reduces the sequencing burden of traditional approaches by encoding unique DNA barcodes within the transposon.^10^ An initial TnSeq experiment maps each barcode to a specific transposon insertion, and then barcodes from subsequent fitness screens are PCR amplified, sequenced, and counted in a process termed “BarSeq”. Thus, RB-TnSeq simplifies sample preparation and multiplexing for high-throughput screening.^10,11^

Although TnSeq enables the examination of many pathways with relatively low experimental effort, increased output introduces a new problem of data resolvability; identification of a few key genes among thousands becomes analytically challenging. Even when thresholding for extreme fitness values (|fitness| > 2) and introducing a *t*-like significance cutoff, RB-TnSeq studies may identify many dozens to hundreds of genes as potential contributors to growth in a condition.^8,12,13^ Metabolic engineers seeking to leverage a small number of critically important genes for modification must narrow the list of candidate genes, but quantitative methods for this process are limited. Thus, pathway-relevant genes from RB-TnSeq are often selected on the basis of *a priori* knowledge of the pathway,^14^ screening of multiple mutants,^12^ or cross-referencing with complementary -omics data sets.^15^

Here, we demonstrate experimental and analytical approaches that streamline the analysis of RB-TnSeq data (Figure 1). Namely, the choice of baseline condition used to inoculate enrichment cultures influences the fitness readout, use of a medium reference distinguishes between general growth effects and those specific to the target enrichment, and use of biological replicates helps resolve statistically significant fitness changes. Additional discussion examines whether transposon insertion counts near gene termini should be excluded from analysis. The examples provided in this study utilize a previously constructed transposon mutant library in *Pseudomonas putida* KT2440 (hereafter, KT2440),^16^ a well-characterized bacterium often employed as a chassis for metabolic engineering and bioproduct generation.^17^ Although the fundamental gene fitness calculations remain unchanged, the adjustments to experimental design and data analysis presented here improve the resolution of RB-TnSeq outputs, providing a strong foundation for further characterization of the organism of interest. Importantly, while many of the examples described here focus on RB-TnSeq data sets in the context of bacterial metabolism, the approaches are generally applicable to many types of TnSeq experiments.

**Figure 1:**
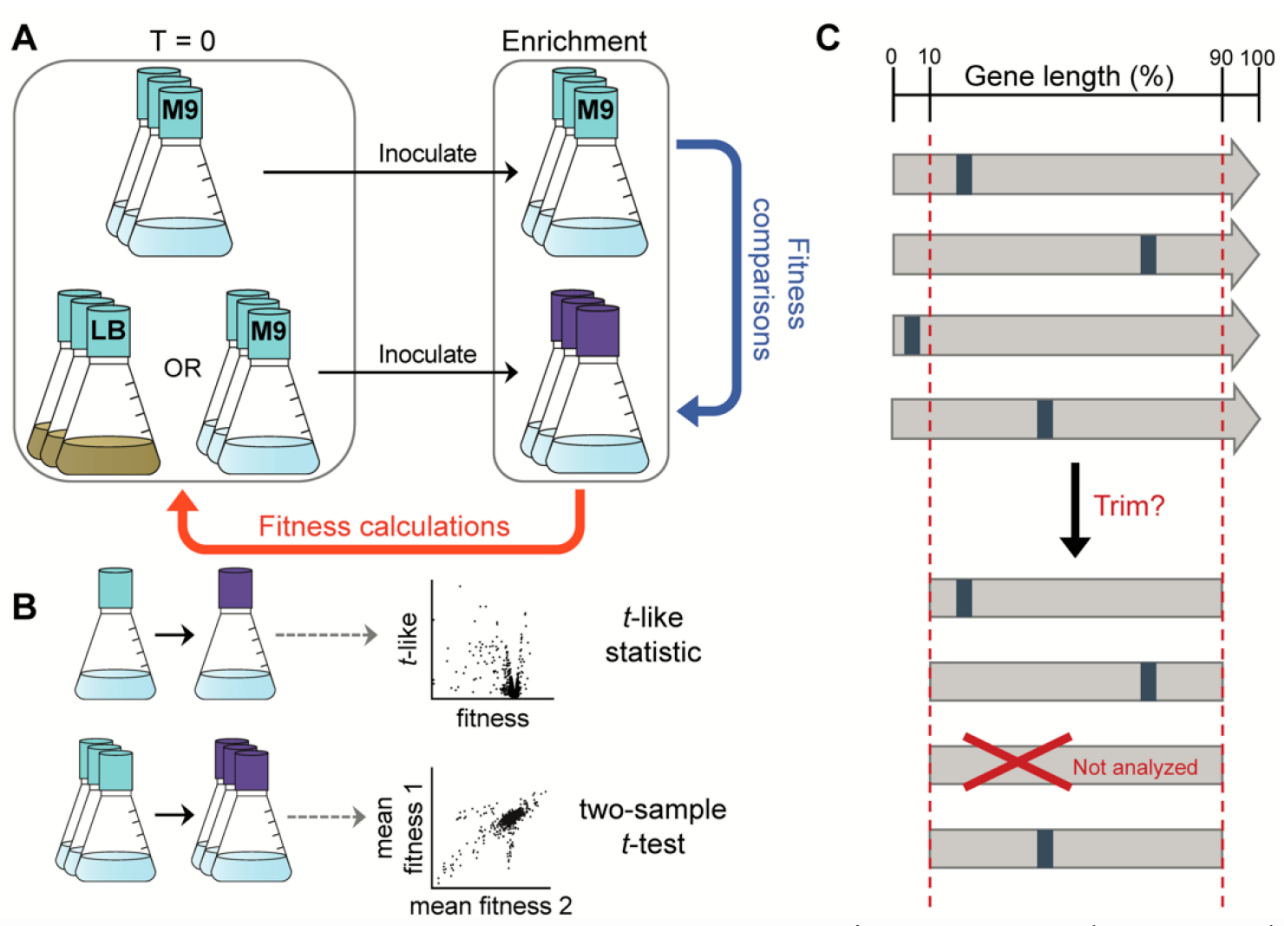
Summary of experimental and analytical considerations for RB-TnSeq. (A) Time-zero (*T* = 0) cultures are used as the “baseline” reference for fitness calculations and may either be grown in rich medium (e.g., LB) or minimal medium (e.g., M9 + glucose), the selection of which influences the fitness calculation between enrichment cultures and reference (*T* = 0) cultures. Enrichment cultures consist of either a passaged medium reference culture (top) or a desired selection condition (bottom). Fitness comparisons may be made between reference and selection enrichment cultures by calculating average fitness scores for each of the two groups. (B) If a practitioner prioritizes high throughput, a single sample may be used for each of the *T* = 0 and enrichment cultures, but this limits the gene fitness confidence metric to only the *t*-like statistic, which can vary between replicates. For increased statistical confidence, biological triplicates enable calculation of mean fitness and a two-sample *t*-score for each gene. (C) In theory, transposon insertions (blue bands) occur randomly along the length of a gene. Some analytical approaches discard genes from analysis if the transposon insertion lies within the first or last 10% of the gene coding sequence length.

## Results and Discussion

### Reference Medium Selection

Selection of an appropriate “baseline” condition to act as a reference for relative quantification of strain abundance following growth selection is critical for all TnSeq experiments. BarSeq analysis employs these count data to determine strain and gene fitness. Typically, the baseline for BarSeq fitness calculations, termed the “time-zero” (*T* = 0) sample, is an aliquot of the library grown to mid log growth phase and used to inoculate the enrichment conditions. Many RB-TnSeq experiments grow *T* = 0 cultures in rich medium (lysogeny broth, LB) supplemented with the antibiotic used for selection of functional transposon insertions (kanamycin).^10,14,18^

Following initial library generation and selection, transposon insertions are stable in the absence of an antibiotic, eliminating the need for further antibiotic selection during regrowth.^19^ Particular care should be taken regarding antibiotic addition for studies with transposons encoding titratable antibiotic resistance systems, such as Tn10,^20^ as variable expression of the antibiotic resistance gene may lead to unintended fitness effects.

**Figure 2:**
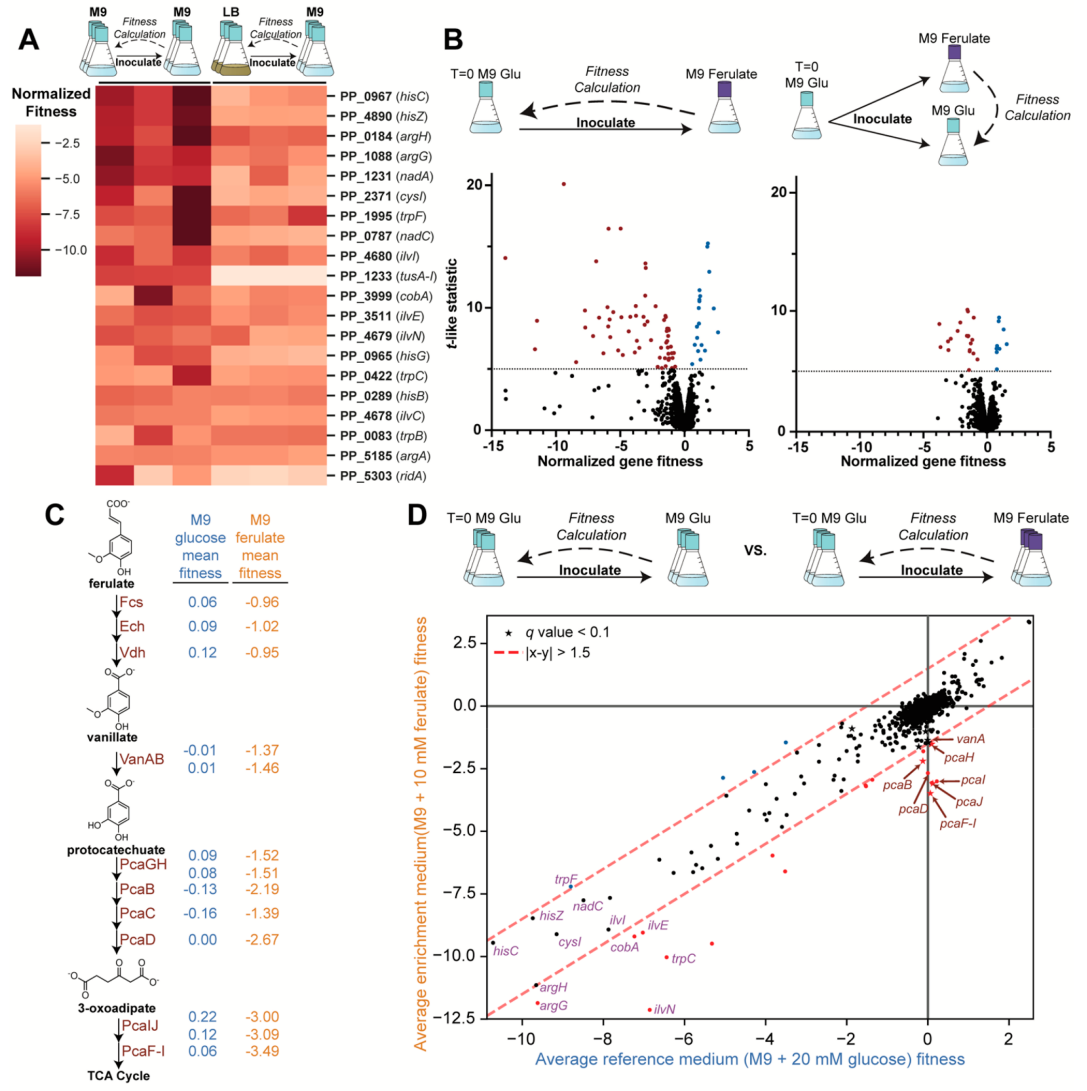
Baseline considerations for fitness calculations during RB-TnSeq experiments. (A) Triplicate *T* = 0 cultures were prepared in M9 + 20 mM glucose and used to directly inoculate M9 + 20 mM glucose enrichment cultures. Another set of triplicate *T* = 0 cultures was prepared in LB and washed once in M9 salts prior to inoculation of M9 + 20 mM glucose enrichment cultures. The 20 genes with lowest mean fitness scores from the M9 *T* = 0 data set are shown with corresponding fitness scores from the LB *T* = 0 data set. (B) Normalized fitness scores and *t*-like statistics plotted for (left) a single M9 + 10 mM ferulate enrichment culture using either the M9 + 20 mM glucose *T* = 0 condition as the baseline or (right) a parallel M9 + 20 mM glucose enrichment culture as the baseline. Significant (*t-*like statistic >5) negative and positive fitness values marked in red or blue, respectively. (C) Normalized fitness values for triplicate M9 + 20 mM glucose or M9 + 10 mM ferulate cultures were calculated using the M9 + 20 mM glucose *T* = 0 condition as the baseline and shown for members of the ferulate catabolism pathway in KT2440. (D) Average normalized fitness values for M9 + 20 mM glucose (reference medium) cultures were compared to those for M9 + 10 mM ferulate (enrichment medium) cultures using the M9 + 20 mM glucose *T* = 0 condition as the baseline. Red dashed lines indicate genes with an average fitness score difference >1.5. Significance was determined with a two-sample *t-*test, where a *q* value <0.1 (stars) denotes a significant fitness disparity between the two conditions.

For metabolism studies, the risk of metabolite carryover from *T* = 0 cultures grown in rich medium is often mediated by removing rich medium and resuspending cells in carbon-free medium prior to enrichment media inoculation, including several intermediate washes with carbon-free medium, at times.^10,14,18^ An alternative is direct inoculation of enrichment cultures from *T* = 0 cultures prepared in minimal medium, which reduces potential damage from shear stress during centrifugation.^21^ To compare fitness outcomes between these approaches, triplicate *T* = 0 cultures were prepared in (i) LB, where cells were pelleted and resuspended in M9 salts, or (ii) M9 + 20 mM glucose, where no pelleting was performed. Each of these preparations was used to inoculate a set of triplicate enrichment cultures in M9 + 20 mM glucose. Inspection of 25 genes with lowest mean fitness in the M9 + 20 mM glucose enrichment revealed that, while fitness trends were maintained with either *T* = 0 baseline condition, fitness defects were, on average, more pronounced when using the minimal medium *T* = 0 baseline (Figure 2A, File S1, Figure S1). Many of these gene disruptions are predicted to be auxotrophic, so metabolite carryover from the LB *T* = 0 samples may have tempered the negative gene fitness outcomes. Additional washes may overcome this effect, but the impact of repeated centrifugation and washing was not examined in this study. Overall, prior studies and the data presented here suggest that potential gains from controlling against metabolite carryover should be weighed carefully against the possibility for centrifugation stress to alter the cell surface composition^22^ and perturb growth behavior^23^ disproportionately for a subset of mutants within the library.

One concern in using minimal medium *T* = 0 cultures is that the lack of nutrients found in rich medium may deplete culture diversity. However, the number of genes analyzed (containing >30 transposon counts per gene) when using M9 + 20 mM glucose *T* = 0 cultures did not significantly differ from the number of genes analyzed when using LB *T* = 0 cultures (Table S1). Rather, sequencing depth seemed to be a better predictor of count diversity, where *T* = 0 samples with fewer sequence reads were more reduced in the number of genes analyzed (Table S1). It is worth noting, bacteria grown in minimal medium can excrete metabolites that will transfer to enrichment cultures during direct inoculation. However, this work demonstrates that these metabolites are less likely to obfuscate fitness defects associated with knockout of conditionally essential genes than those from LB-derived cultures (Figure S1).

Together, these data suggest that removal of rich medium without intermediate wash steps can contribute fitness-dampening effects. In instances where metabolite carryover from inoculation cultures is undesirable and/or practitioners wish to avoid centrifugal stress, direct inoculation of cultures prepared in minimal medium offers one possible solution. Ultimately, these considerations should be combined with the hypotheses being tested and the nutritional requirements of the host organism to select an appropriate baseline condition for TnSeq.

### Utility of a Medium Reference

TnSeq analysis usually calculates gene fitness as a ratio of strain counts in the enrichment condition relative to the *T* = 0 condition; however, this approach provides output where gene disruptions specifically influencing fitness for the targeted physiological process and those general to growth cannot be easily distinguished. This phenomenon was demonstrated by growing the KT2440 transposon library in conditions relevant to ferulate catabolism. A single replicate of the library was enriched in M9 + 10 mM ferulate, and fitness values were calculated using the M9 + 20 mM glucose *T* = 0 baseline (Figure 2B, File S1). Of the 62 genes with significant negative fitness (*t*-like statistic >5), 11 (18%) were associated with ferulate catabolism (Figure 2C), with many other genes encoding essential amino acid and vitamin biosynthesis pathways. When the same analysis was performed using a passaged medium reference (i.e., a M9 + 20 mM glucose enrichment) as the baseline, only 20 genes showed significant negative fitness, and 11 (55%) of these were the same ferulate catabolism genes identified previously (Figure 2B, File S1). Moreover, many of the previously significant amino acid and vitamin biosynthesis genes were no longer deemed significant or eliminated from the data set altogether, demonstrating that mutants with auxotrophies that do not necessarily influence catabolism of the target metabolite can exhibit generally reduced fitness during growth in minimal medium. Therefore, use of a medium reference, instead of a *T* = 0 sample, as the baseline for fitness calculations can effectively resolve genes with enrichment-specific roles in metabolism.

One caveat to the parallel medium reference approach is that information regarding generally beneficial or harmful disruptions is lost. In some studies, enrichment-specific fitness effects are visually resolved from those shared between conditions by plotting enrichment fitness scores (relative to a *T* = 0 baseline) against each other.^10,18^ In these plots, shared fitness effects fall roughly along the *Y* = *X* line of origin. Average gene fitness scores for the KT2440 library grown in M9 + 20 mM glucose (passaged medium reference) were compared to those in M9 + 10 mM ferulate (enrichment condition), using the M9 + 20 mM glucose *T* = 0 cultures as the baseline (Figure 2D, File S1). Accordingly, ferulate catabolism genes, which displayed negative fitness during ferulate enrichment and little fitness change in the glucose reference condition (Figure 2C), fell along the *X* = 0 line. Genes with fitness scores that deviated significantly from 0 but fell along the *Y* = *X* line of origin contributed a general fitness effect in both conditions. As expected, genes with general negative fitness effects included those for amino acid and vitamin biosynthesis, which are essential in growth conditions where these metabolites are absent.

### Biological Replicates

For all TnSeq applications, fitness comparisons can be drawn between many conditions by using single replicates of different enrichment conditions. However, fitness scores between replicates may show a large degree of variation, especially those with very negative fitness scores (Figure 2A). Therefore, it may be desirable to prioritize resolution over throughput by using several biological replicates. In this case, a two-sample *t*-test with multiple testing correction (*q* value <0.1) can quantify confidence in fitness differences between two groups, such as medium reference and enrichment replicates (see Materials and Methods). In KT2440, this approach demonstrated that mean fitness differences for many genes with severe fitness defects in Figure 2D were not statistically significant, enabling confident identification of ferulate catabolic genes. Therefore, for situations where definitive identification of a physiological process or pathway is desirable, use of multiple replicates can improve the resolution of the RB-TnSeq experiment. For scenarios that demand higher throughput across many conditions, single replicates may be sufficient to identify particularly strong gene fitness “hits” for subsequent validation.

### Transposon Insertion Count Exclusion

Regardless of transposon type or host organism, transposon insertions localized near gene termini might not completely abrogate gene expression. For this reason, there exists significant disparity in whether to trim data sets based upon transposon localization, whether to exclude transposon counts only at the 3′ end or both ends, and how close to the end of a gene is an appropriate cutoff for exclusion.^9,10,24−26^ Indeed, one study examining count trimming in detail found that removing counts in the last 10% of a gene only marginally changed fitness for four genes.^9^ This work aimed to further explore the utility of transposon count trimming by examining fitness in the KT2440 transposon library after enrichment in M9 + 10 mM ferulate. Gene fitness values were calculated with and without trimming of insertion counts localized within the first and/or last 10% of a gene (Table 1). Once again, this example is provided in the context of metabolism, but since count trimming is common across all TnSeq applications, these findings apply generally.

**Table 1:** Effect of Trimming on Fitness Calculations

	nontrimmed
genes analyzed^a^	4937

	trim first 10%	trim last 10%	trim both ends (10% each)
genes affected by trimming^b^	3005	3225	4139
genes eliminated from analysis	65	153	251
genes with a fitness change > |1|	5	13	17

a Genes analyzed contain fitness data from all three biological replicates.

b Genes from the nontrimmed data set that contained counts in the trimmed region.

Overall, several genes failed to meet the count threshold for fitness analysis when counts were excluded for transposons localized to the first 10% (65 genes), last 10% (153 genes), or 10% on both ends (251 genes) (Table 1, File S1). However, only a handful of the genes affected by trimming showed mean fitness changes > |1|, between the trimmed and nontrimmed data sets (Table 1, File S1). Interestingly, two loci (PP_5289 and PP_5185) showed conflicting fitness changes, depending on the trimming method used (Figure S2). Additionally, trimming 5% from both ends showed fitness differences > |1| only for two genes, as compared to trimming 10% from both ends (Figure S2). These findings support the assertion that trimming insertion counts from gene ends may be unnecessary for identification of conditionally essential genes and detrimental if it substantially reduces the number of genes analyzed. For libraries with dense, evenly distributed transposon insertion coverage, trimming is not likely to result in significant gene fitness changes. However, if trimming is desired, various cutoffs can be tested to identify data-specific thresholds that do not significantly reduce analyzed genes. Importantly, the *mariner* transposon used in the KT2440 library does not contain an outward-facing promoter, but in-frame insertion of a transposon with an outward-facing promoter in a gene’s early 5′ end could allow continued expression of functional protein, so transposon selection is critical.^27,28^

Beyond identifying conditionally essential genes, RB-TnSeq is useful for identification of “absolute” essential genes that are critical for growth in any condition (e.g., encoding ribosomal subunits, t-RNAs, etc.). Observation of genes with a complete absence of transposon insertions is a common technique for identification of candidate essential genes,^29^ and this approach successfully identified many essential gene candidates from the KT2440 transposon library with known essentiality in other pseudomonads (File S1).^30^ Interestingly, the list of genes eliminated from analysis following trimming contained a number of genes with predicted essentiality (DNA gyrase subunits, t-RNAs, etc.), showing that end-trimming may still prove useful for identifying “absolute” essentiality (File S1). Other applications of TnSeq may also require end trimming, as in methods for essential protein domain discovery.^31^

## Conclusions

TnSeq experiments cover a wide range of fields and applications, but this study provides a set of core principles that should be considered before embarking on any TnSeq campaign. The examples provided here demonstrate that experimental design and selection of analytical approaches can substantially influence data resolution, so practitioners are encouraged to examine each component in the context of their specific host organism and scientific question.

## Materials and Methods

### Media Preparation

KT2440 was maintained on lysogeny broth (LB; Lennox) broth (Sigma-Aldrich). For sole carbon and energy source experiments, KT2440 was cultivated in M9 minimal medium (6.78 g/L Na_2_HPO_4_, 3 g/L KH_2_PO_4_, 0.5 g/L NaCl, 1 g/L NH_4_Cl, 2 mM MgSO_4_, 100 μM CaCl_2_, and 18 μM FeSO_4_) with carbon sources added as indicated in Table S2. All cultures were incubated at 30 °C with shaking at 225 rpm. All chemicals were obtained from Sigma-Aldrich.

### Transposon Library Experiments

Construction of a randomly barcoded *mariner* transposon insertion library in KT2440 was previously described.^16^ This library contains 185 401 uniquely barcoded transposon insertions with 32 591 insertions mapping to intergenic regions. The remaining 152 810 insertions mapped to 5213 of 5661 *P. putida* KT2440 genes across the 6 181 873 bp genome. A list of genes without any transposon insertions is provided in File S1. Six 1 mL library aliquots, previously grown in LB + 50 μM kanamycin to an optical density at 600 nm (OD_600 nm_) of ∼1.0, were thawed on ice. For *T* = 0 cultures (experiment 1 and 2, Table S2), 125 mL baffled flasks containing 25 mL M9 + 20 mM glucose or 25 mL LB medium were inoculated with one aliquot of the thawed library, resulting in triplicate cultures of each. *T* = 0 cultures were incubated until OD_600 nm_ ∼ 1.0. Cells from *T* = 0 cultures were used to inoculate enrichment cultures (experiments 3–6, Table S2) to an initial OD_600 nm_ of 0.02. Cells from the M9 + glucose *T* = 0 culture were not washed prior to inoculation, but cells from the LB *T* = 0 culture were centrifuged for 1 min at 10 000*g* and resuspended in an equal volume of 1× M9 salts prior to inoculation. Enrichment cultures were incubated until the OD_600 nm_ reached ∼1.0 (Table S2). Optical density measurements were taken on a Beckman DU 640 spectrophotometer, using relevant sterile medium as a blank. When cultures reached the desired cell density, a 1 mL aliquot was withdrawn from each flask and centrifuged at 10 000*g* for 1 min, and then pellets were frozen at −80 °C. Pellets were collected for all *T* = 0 cultures as well as all passaged cultures.

### BarSeq

Cell pellets from each transposon library experiment were thawed on ice and genomic DNA (gDNA) was isolated from each using the GeneJet Genomic DNA Purification Kit (Thermo Scientific). BarSeq PCR reactions were performed as previously described,^10^ using a common reverse primer (BarSeq_P1) and one of 18 forward primers encoding a unique sequence used for demultiplexing sequence reads (BarSeq_P2_ITXXX, Table S3). Each reaction was performed in 15 μL total volume, using Q5 High-Fidelity 2× Master Mix (New England Biolabs), 0.5 μM of each primer, 75 ng template gDNA, and 2% v/v DMSO. Thermal cycles were performed as follows: (i) 98 °C, 4 min, (ii) 25 cycles of the following: 98 °C, 30 s; 55 °C, 30 s; 72 °C, 30 s, (iii) 72 °C, 5 min. To verify the presence of BarSeq PCR products, 4 μL of each reaction was run on a 1% w/v agarose gel and a band ∼180 bp in size was confirmed. The sequencing pool was generated by combining 8 μL of each PCR product in a single tube and treating with DpnI enzyme (New England Biolabs, USA) using 1 unit DpnI/4.5 μL PCR reaction for 30 min at 37 °C. The entire pool was run on a 1% w/v agarose gel, and the band at ∼180 bp was excised and purified using the Zymoclean Gel DNA Recovery Kit (Zymo Research). The sample was sequenced on an Illumina HiSeq instrument with 2 × 150 bp reads (Azenta Life Sciences), and samples were demultiplexed according to their BarSeq P2 indices. Number of reads and quality information for each sample can be found in Table S4. Sequencing data (fastq files) were deposited at the NCBI Sequence Read Archive (SRA) with accession number PRJNA809672.

### Calculation of Gene Fitness

BarSeq reads were initially analyzed using a series of previously described Perl scripts.^10,16^ First, reads were tabulated according to the number of times each barcode was seen in each sample (MultiCodes.pl). The table of barcode counts was then merged with a table of previously defined genomic barcode locations in the KT2440 library (combineBarSeq.pl; genomic insertions table from http://morgannprice.org/FEBA/Putida/pool). The output of these processing steps, “all.poolcount”, tabulated strain counts for each transposon insertion across all samples (File S1). All samples successfully passed quality control metrics as defined by the Perl scripts. Strain and gene fitness calculations were performed as previously described,^10^ using custom Python scripts (https://github.com/beckham-lab/RB-TnSeq). Transposon insertion counts were not trimmed from gene ends (unless stated otherwise) and fitness normalization used only the approach described for small gene scaffolds, since scaffold size was not well-defined in the previous work.^10^ Briefly, transposon insertion counts were used to determine strain fitness calculated as a normalized log_2_ ratio of barcode reads in the enrichment sample vs the baseline sample. Gene fitness was calculated as the weighted average of the strain fitness for all transposon insertions at that locus and normalized by subtracting the median unnormalized fitness within a 251 gene sliding window. Transposon counts were excluded if three reads/strain were not present in the baseline condition, and if a gene did not contain >30 transposon insertion reads in the baseline condition, it was excluded from analysis. For statistical analysis using data without multiple replicates, a *t*-like statistic was calculated for each gene as previously described,^10^ and genes with a *t-*like statistic > |5| were considered significant. For statistical analysis using data with three biological replicates, comparison of mean fitness values between enrichment and medium reference culture groups was facilitated by a two-sample *t*-test, where the *p* value was corrected for multiple testing via the positive false discovery rate (pFDR) method.^32,33^ The corrected *p* value (*q* value) is calculated as *q*_*i*_ = *p*_*i*_ × *N*/*i*, where *p*_i_ is the *i*-th smallest *p* value out of *N* total *p* values. Since this equation can yield higher *q* values for records with lower *p* values, the pFDR *q* values were then adjusted for monotonicity, where q*_i_ is the adjusted *q* value, and its value is set as the smallest uncorrected *q* value *q*_*k*_, *k* ≥ *i*.^34^ Both unadjusted and adjusted *q* values are reported. Gene data were excluded if fitness values were not obtained for all three biological replicates from both conditions.

## Supporting Information Available

File S1: Raw transposon count and fitness data used in Figure 2 and Table 1 (XLSX)
Table S1: Genes in each *T* = 0 sample that meet the >30 count cutoff for fitness analysis; Table S2: Experimental layout description; Table S3: BarSeq primers used in this study; Table S4: NGS count and quality metrics; Figure S1: Fitness comparison for the M9 + 20 mM glucose enrichment condition using LB or M9 + 20 mM glucose grown cultures; Figure S2: Fitness comparison for the M9 + 10 mM ferulate enrichment condition using nontrimmed or end-trimmed data (PDF)

## Abbreviations

KT2440: *Pseudomonas putida* KT2440
RB-TnSeq: randomly barcoded transposon insertion sequencing
LB: lysogeny broth
*T* = 0: time-zero.
