# Supplementary material for: Experimental and Analytical Approaches for Improving the Resolution of Randomly Barcoded Transposon Insertion Sequencing (RB-TnSeq) Studies
Source: ACS Synth Biol. 2022 Jun 3;11(6):2015–21. doi: 10.1021/acssynbio.2c00119 (PMC9208016; doi:10.1021/acssynbio.2c00119)
Supplement: Supplementary file 2 — sb2c00119_si_002.pdf [file sb2c00119_si_002.pdf]

Supplementary Information for:

Experimental and analytical considerations for improving the resolution of randomly barcoded transposon insertion sequencing (RB-TnSeq) studies

Andrew Borchert,[1,2*] Alissa Bleem,[1,2*] and Gregg T. Beckham[1,2#]

[1]Renewable Resources and Enabling Sciences Center, National Renewable Energy Laboratory, Golden CO, United States
[2]Center for Bioenergy Innovation, Oak Ridge National Laboratory, Oak Ridge TN 37830, USA
*Denotes equal contribution
#Corresponding author: Gregg.Beckham@nrel.gov

Table S1. Summary of genes that passed the baseline cutoff (>30 gene counts) required for fitness analysis. The number of NGS reads associated with each sample is also provided.

Experiment / Replicate	Condition description	Genes included in analysis	# Reads
1A	M9 + 20 mM D-glucose (T=0)	4953	5,618,453
1B	M9 + 20 mM D-glucose (T=0)	5022	8,197,689
1C	M9 + 20 mM D-glucose (T=0)	5004	7,186,676
2A	LB (T=0)	5015	7,514,940
2B	LB (T=0)	4997	7,338,799
2C	LB (T=0)	5024	8,310,257

Table S2. Experimental layout description with corresponding OD_{600} readings taken at time of sampling. Values A, B, and C denote the biological replicate.

Experiment	Media composition and description	OD_{600nm}_A	OD_{600nm}_B	OD_{600nm}_C
1	M9 + 20 mM D-glucose (T=0)	1.153	0.900	0.990
2	LB (T=0)	1.087	1.265	1.075
3	M9 + 20 mM D-glucose (passaged from #1)	1.002	1.090	1.252
4	M9 + 20 mM D-glucose (passaged from #2)	0.984	0.944	1.078
5	M9 + 10 mM ferulate (passaged from #1)	1.056	0.910	1.285

Table S3. BarSeq forward primers, BarSeq_P2_ITXXX, used in this study (original sequences from Wetmore *et al.*[1]). BarSeq P2 indices used for demultiplexing sequencing data are in bold.

Experiment/ Replicate	Index name	Index sequence	BarSeq_P2 primer sequence
1A	IT001	ATCACG	CAAGCAGAAGACGGCATACGAGAT**CGTGAT**GTGACTGGAGTTCAGACGTGTGCTCTTCCGATCTGATGTCCACGAGGTCTCT
1B	IT002	CGATGT	CAAGCAGAAGACGGCATACGAGAT**ACATCG**GTGACTGGAGTTCAGACGTGTGCTCTTCCGATCTGATGTCCACGAGGTCTCT
1C	IT003	TTAGGC	CAAGCAGAAGACGGCATACGAGAT**GCCTAA**GTGACTGGAGTTCAGACGTGTGCTCTTCCGATCTGATGTCCACGAGGTCTCT
2A	IT004	TGACCA	CAAGCAGAAGACGGCATACGAGAT**TGGTCA**GTGACTGGAGTTCAGACGTGTGCTCTTCCGATCTGATGTCCACGAGGTCTCT
2B	IT005	ACAGTG	CAAGCAGAAGACGGCATACGAGAT**CACTGT**GTGACTGGAGTTCAGACGTGTGCTCTTCCGATCTGATGTCCACGAGGTCTCT
2C	IT006	GCCAAT	CAAGCAGAAGACGGCATACGAGAT**ATTGGC**GTGACTGGAGTTCAGACGTGTGCTCTTCCGATCTGATGTCCACGAGGTCTCT
3A	IT007	CAGATC	CAAGCAGAAGACGGCATACGAGAT**GATCTG**GTGACTGGAGTTCAGACGTGTGCTCTTCCGATCTGATGTCCACGAGGTCTCT
3B	IT008	ACTTGA	CAAGCAGAAGACGGCATACGAGAT**TCAAGT**GTGACTGGAGTTCAGACGTGTGCTCTTCCGATCTGATGTCCACGAGGTCTCT
3C	IT009	GATCAG	CAAGCAGAAGACGGCATACGAGAT**CTGATC**GTGACTGGAGTTCAGACGTGTGCTCTTCCGATCTGATGTCCACGAGGTCTCT
4A	IT010	TAGCTT	CAAGCAGAAGACGGCATACGAGAT**AAGCTA**GTGACTGGAGTTCAGACGTGTGCTCTTCCGATCTGATGTCCACGAGGTCTCT
4B	IT011	GGCTAC	CAAGCAGAAGACGGCATACGAGAT**GTAGCC**GTGACTGGAGTTCAGACGTGTGCTCTTCCGATCTGATGTCCACGAGGTCTCT
4C	IT012	CTTGTA	CAAGCAGAAGACGGCATACGAGAT**TACAAG**GTGACTGGAGTTCAGACGTGTGCTCTTCCGATCTGATGTCCACGAGGTCTCT
5A	IT019	GTGAAA	CAAGCAGAAGACGGCATACGAGAT**TTTCAC**GTGACTGGAGTTCAGACGTGTGCTCTTCCGATCTGATGTCCACGAGGTCTCT
5B	IT020	GTGGCC	CAAGCAGAAGACGGCATACGAGAT**GGCCAC**GTGACTGGAGTTCAGACGTGTGCTCTTCCGATCTGATGTCCACGAGGTCTCT
5C	IT021	GTTTCG	CAAGCAGAAGACGGCATACGAGAT**CGAAAC**GTGACTGGAGTTCAGACGTGTGCTCTTCCGATCTGATGTCCACGAGGTCTCT

Table S4. Sequencing read counts and quality metrics for each sample used in this work.

Experiment / Replicate	# Reads	Yield (Mbp)	Mean Quality Score
1A	5,618,453	1686	32.41
1B	8,197,689	2459	32.58
1C	7,186,676	2156	32.14
2A	7,514,940	2254	32.47
2B	7,338,799	2202	32.47
2C	8,310,257	2493	32.56
3A	7,105,842	2132	32.63
3B	6,731,403	2019	32.53
3C	6,202,795	1861	32.52
4A	7,596,456	2279	32.46
4B	6,943,381	2083	32.3
4C	6,937,674	2081	32.47
5A	7,144,571	2143	32.56
5B	6,860,069	2058	32.35
5C	5,727,812	1718	32.4

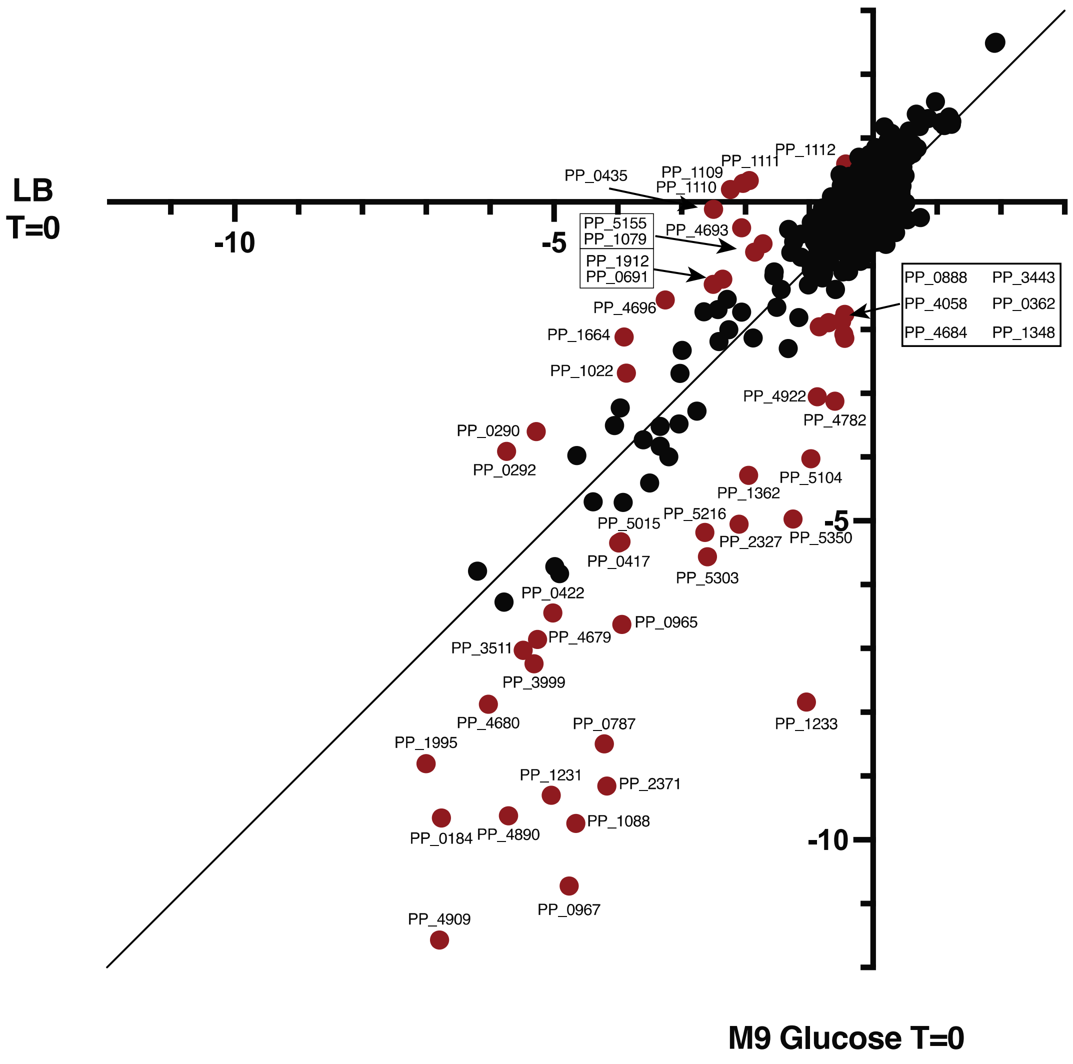

Figure S1. Fitness comparison for the M9 + 20 mM D-glucose enrichment condition using LB or M9 + 20 mM D-glucose inoculum (T=0) cultures. LB-derived mean fitness values are plotted on the X-axis and mean fitness derived from M9 + 20 mM D-glucose are plotted on the y-axis. Means values are taken from three biological replicates and genes with a mean fitness difference >|1| between the datasets have red markers and are labeled with locus tags.

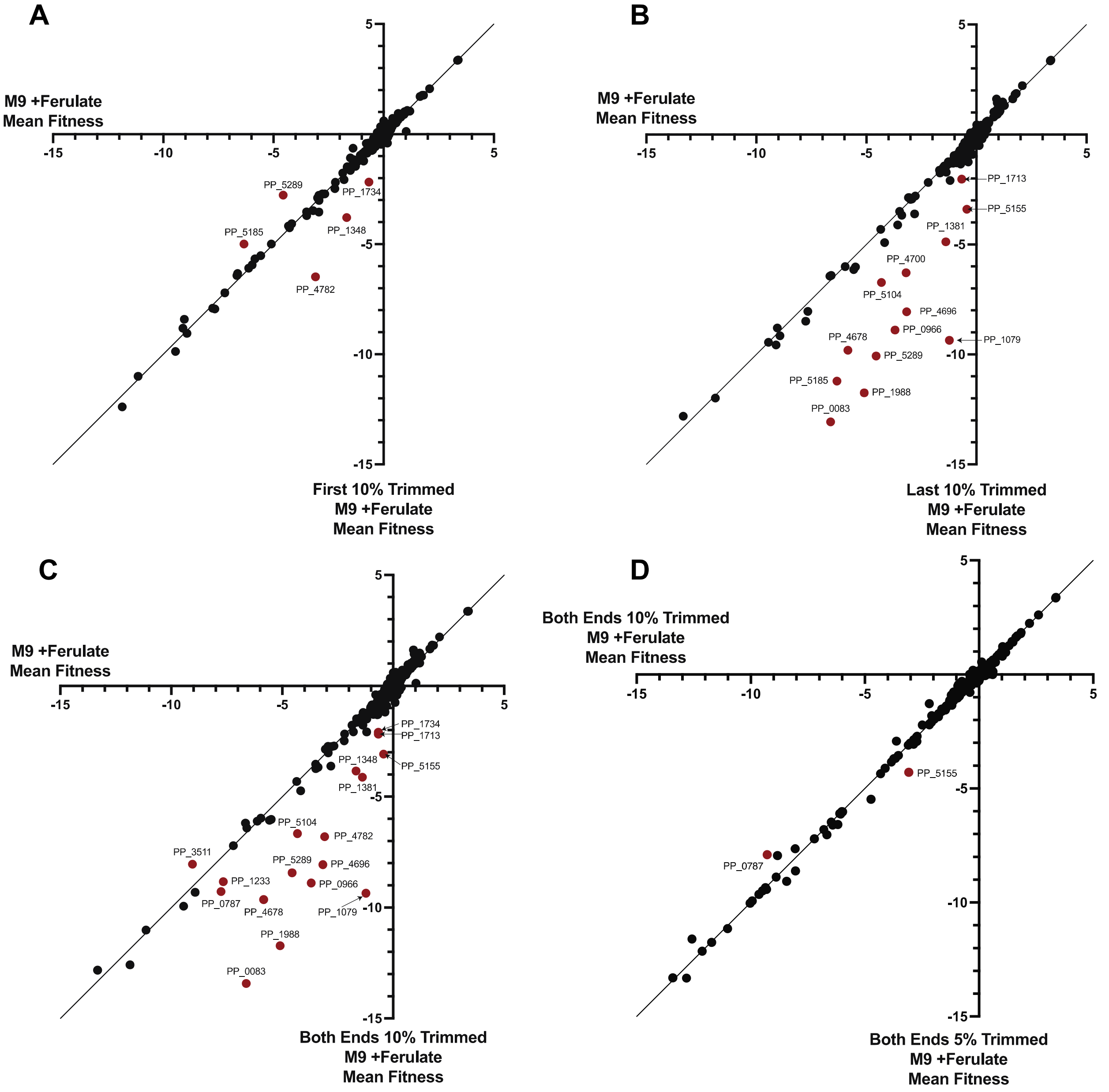

Figure S2. Fitness comparison for the M9 + 10 mM ferulate enrichment condition where (**A-C**) mean fitness derived from three biological replicates using non-trimmed data is plotted on the X-axis and mean fitness derived from three biological replicates using excluding counts from transposons localized within (**A**) the first 10% of a gene's coding DNA sequence (CDS), (**B**) the last 10% of a gene's CDS, or (**C**) the first and last 10% of a gene's CDS. (**D**) mean fitness derived from three biological replicates excluding counts from transposons localized with the first and last 10% of a gene's CDS on the X-axis vs. the first and last 5% of a gene's CDS. Genes with a mean fitness difference >|1| between the trimmed dataset and the non-trimmed dataset have red markers and are labeled with locus tags. No differences were statistically significant from the non-trimmed dataset using any of the trimming approaches (q value < 0.1) (**File S1**).[2,3]

REFERENCES

(1) Wetmore, K. M.; Price, M. N.; Waters, R. J.; Lamson, J. S.; He, J.; Hoover, C. A.; Blow, M. J.; Bristow, J.; Butland, G.; Arkin, A. P. Rapid quantification of mutant fitness in diverse bacteria by sequencing randomly bar-coded transposons. *mBio* **2015**, *6* (3), e00306-00315. DOI: 10.1128/mBio.00306-15.

(2) Benjamini, Y.; Hochberg, Y. Controlling the false discovery rate: A practical and powerful approach to multiple testing. *J. Roy. Stat. Soc. Ser. B. (Stat. Method.)* **1995**, *57* (1), 289-300. DOI: 10.1111/j.2517-6161.1995.tb02031.x.

(3) Storey, J., D. The positive false discovery rate: a Bayesian interpretation and the *q*-value. *Ann. Stat.* **2003**, *31* (6), 2013-2035. DOI: 10.1214/aos/1074290335.
